# Challenges Women with Disability Face in Accessing and Using Maternal Healthcare Services in Ghana: A Qualitative Study

**DOI:** 10.1371/journal.pone.0158361

**Published:** 2016-06-27

**Authors:** John Kuumuori Ganle, Easmon Otupiri, Bernard Obeng, Anthony Kwaku Edusie, Augustine Ankomah, Richard Adanu

**Affiliations:** 1 Department of Population, Family and Reproductive Health, School of Public Health, University of Ghana, Legon, Accra, Ghana; 2 Department of Population, Family and Reproductive Health, Kwame Nkrumah University of Science and Technology, Kumasi, Ghana; 3 Department of Sociology and Social Work, Faculty of Social Sciences, Kwame Nkrumah University of Science and Technology, Kumasi, Ghana; 4 Centre for Disability and Rehabilitation Studies, School of Medical Sciences, Kwame Nkrumah University of Science & Technology, Kumasi, Ghana; Nathan Kline Institute and New York University School of Medicine, UNITED STATES

## Abstract

**Background:**

While a number of studies have examined the factors affecting accessibility to and utilisation of healthcare services by persons with disability in general, there is little evidence about disabled women's access to maternal health services in low-income countries and few studies consult disabled women themselves to understand their experience of care and the challenges they face in accessing skilled maternal health services. The objective of this paper is to explore the challenges women with disabilities encounter in accessing and using institutional maternal healthcare services in Ghana.

**Methods and Findings:**

A qualitative study was conducted in 27 rural and urban communities in the Bosomtwe and Central Gonja districts of Ghana with a total of 72 purposively sampled women with different physical, visual, and hearing impairments who were either lactating or pregnant at the time of this research. Semi-structured in-depth interviews were used to gather data. Attride-Stirling’s thematic network framework was used to analyse the data. Findings suggest that although women with disability do want to receive institutional maternal healthcare, their disability often made it difficult for such women to travel to access skilled care, as well as gain access to unfriendly physical health infrastructure. Other related access challenges include: healthcare providers’ insensitivity and lack of knowledge about the maternity care needs of women with disability, negative attitudes of service providers, the perception from able-bodied persons that women with disability should be asexual, and health information that lacks specificity in terms of addressing the special maternity care needs of women with disability.

**Conclusions:**

Maternal healthcare services that are designed to address the needs of able-bodied women might lack the flexibility and responsiveness to meet the special maternity care needs of women with disability. More disability-related cultural competence and patient-centred training for healthcare providers as well as the provision of disability-friendly transport and healthcare facilities and services are needed.

## Introduction

Disability may be defined as the consequence of an impairment that may be physical, cognitive, mental, sensory, emotional, developmental, or some combination of these that result in restrictions on an individual's ability to participate in what is considered "normal" in their everyday society [1]. Globally, persons with disability constitute 15% of the world's population [1]. Although the United Nations’ Convention on the Rights of Persons with Disabilities guarantees persons with disabilities the same level of right to access quality and affordable healthcare, including sexual and reproductive healthcare services, as persons without disability [2], such persons are still one of the most marginalised and socially excluded groups in many countries including Ghana [3,4]. This disadvantage transcends several spheres. For example, persons with disabilities have generally poorer health, lower education achievements, fewer economic opportunities and higher rates of poverty than persons without disabilities [1]. In particular, women with disability are more likely to be poorer and have lower social and economic status than their counterparts who have no disability [3,4]. In the context of reproductive health, women with disabilities have largely been ignored in reproductive health research and programming [5]. For example, one recent review on disability in sexual and reproductive health policies and research in Ghana concluded that persons with disabilities have received little attention [6]. Part of the reason for this neglect is that they are often thought not to be sexually active, and less likely to marry or to have children than women without disability [7]. The perception that women with disability are not sexually active has often resulted in limited access to sexual and reproductive health services for them. As recent studies show, access to sexual and reproductive health services by women with disability is still a serious challenge [5,7]. A number of studies have suggested negative social attitudes and cultural assumptions such as the equation of sexuality to being normal and not disabled, physically inaccessible health facilities, insensitivity of healthcare providers, limited knowledge by healthcare providers about disability, and limited information tailored to their health needs as factors hampering access to skilled reproductive and maternal healthcare services by women with disabilities [5, 7, 8–10].

Increasingly, there is a growing body of work in high-income countries that address the health needs, challenges and barriers to prenatal and reproductive care by women with disabilities [5,11]. In low-income settings however, there is paucity of research that asks women with disability about the barriers they encounter in accessing and using maternal healthcare services [5]. In Ghana for example, a number of recent studies have explored various disability issues, including disability culture in Ghana [12], the challenges and opportunities of implementing the World Report on Disability [13], and the inclusion of disability in sexual and reproductive health policies and research in Ghana [6]. Notwithstanding this growing body of research, we are not aware of any studies in Ghana that have examined the problems women with disability encounter when accessing and using maternal healthcare services. The objective of this study is to explore the challenges women with disability in Ghana face in accessing and using maternal healthcare services. To achieve this objective, qualitative data generated from semi-structured interviews with women with disability in Ghana, are used.

### Disability in Ghana

Data from Ghana’s 2010 Population and Housing Census suggest that some 737,743 (3% of the population) persons were living with disability in Ghana [14]. However, other recent estimates suggest Ghana’s disability rate to be between 7–12%, approximately 1.55–2.2 million people [1]. A total of 64% of Ghanaian adults with disabilities are women [13]. The disability prevalence rate among women and men is 10.6% and 6.2% respectively [13]. The three most prevalent types of disability are those related to physical disabilities, visual impairment, and hearing impairment [1].

Ghana is a signatory to the United Nations’ Convention on the Rights of Persons with Disabilities, and in furtherance of this, Ghana enacted the Persons with Disability Act (Act 715) in 2006 [15]. While widespread implementation and enforcement of the act is yet to be reported, the Act guarantees persons with disability the right to access the same or specialised range, quality and standard of healthcare as provided to other persons, including those in the area of sexual and reproductive health [15]. The Act enjoins Ghana’s Ministry of Health to make provisions for free general and specialist medical care, rehabilitative treatment and appropriate assistive services for persons with disability [15]. The Act also mandates Ghana’s Ministry of Health to include the study of disability and related issues in the curricula of training institutions for health professionals to develop appropriate human resources to provide general and specialised rehabilitation services [15]. Other relevant provisions include access to public places and services as well as integration of the needs of persons with disability into the design, construction and operation of transport network [15].

### Maternal health in Ghana

Although Ghana has made progress over the last several decades to improve maternal health, maternal mortality is a serious public health concern. According to the World Health Organization’s most recent estimates, Ghana’s maternal mortality ratio stands at 380 per 100,000 live births [16]. Maternal mortality accounts for 14% of deaths among females aged 12–49 years, and is the second largest cause of female mortality after infectious diseases among women of childbearing age [17]. Despite the fact that Ghana has since 2003 implemented a free maternal healthcare policy, more than 45% of births still occur at home without any form of skilled care in parts of Ghana [18]. In addition, large and growing gradients of inequalities in skilled care services accessibility and utilisation have been observed in Ghana [17,19,20].

## Methods

### Study design

The data reported in this paper are part of a larger multi-methods study that was conducted between November 2012 and May 2015 to examine the effects of Ghana’s free maternal healthcare policy on maternal healthcare access, women’s maternity care seeking experience, equity of access, and barriers to accessibility to and utilisation of maternal and newborn care services. The design of this larger study involved analyses of a nationally representative retrospective household survey data in combination with qualitative research using focus group discussions, in-depth interviews, case studies, and structured field observations as data collection methods. The study was conducted in a total of 27 randomly selected communities in two districts. Participants in the study comprised 257 expectant and lactating mothers, 15 traditional birth attendants, and 20 healthcare providers, including community health nurses, midwives, doctors, health facility managers, district and regional public health nurses, district and regional directors of health, and policy makers at the Ministry of health and Ghana Health Services.

In this paper, the focus is on reporting findings from a sub-sample of the qualitative component of the larger study in which semi-structured in-depth interviews were used to explore the challenges women with disabilities encounter in accessing and using institutional maternal healthcare services in Ghana. Accessibility in this context is defined as a measure of the opportunity to obtain healthcare when it is wanted or needed, while utilisation is the ‘proof of access’ or the actual entry of a given individual or population group into the healthcare delivery system [21].

### Research participants and sampling

As noted above, empirical research was conducted in 27 communities (19 rural and 8 urban) in the Bosomtwe and Central Gonja districts of the Ashanti and Northern regions of Ghana respectively. These two districts were purposively selected to represent northern and southern Ghana in our study. We purposively sampled 72 women with different physical, visual, speech and hearing impairments who were either lactating or pregnant at the time of this research (November 2012 to May 2015). We excluded women with intellectual disabilities partly because of the complexities involved in assessing mental disability and partly because of the research team’s limited knowledge in undertaking such assessment. The women included in the study were identified through screening. An adapted screening tool from the Washington Group on Disability Statistics was used [5]. This screening tool has been successfully used in other low-income contexts to screen and identify women with disabilities [5]. The tool has 35 questions to detect epilepsy, physical, sensory, behavioural, and social function and communication disabilities based on the International Classification of Functioning, Disability and Health (ICF) [22]. The screening tool captures severity of disability by asking respondents to rank their status on a four-point Likert scale [5]. To facilitate easy understanding by participants, the screening tool was translated into the three dominant local dialects–*Twi*, *Dagbani* and *Gonja*–of the study communities. Women were screened at different locations, including their homes, healthcare facilities, market places, and churches/mosques. Women who were either lactating or pregnant at the time of this research and who were identified during the screening process to have any physical, visual, speech and hearing impairment(s) were included in the study. The recruitment process continued until saturation was attained in the data. Community-based surveillance volunteers were recruited and trained to help with the screening and conduct of interviews. These are community members who have been recruited and trained by the Ghana Health Service in various aspects of community health, including but not limited to reporting the outbreak of diseases as well as births and deaths in their communities [23].

### Data collection and analysis

Semi-structured in-depth interviews were used to collect data. A semi-structured interview guide was first developed by one of the research team members (AKE) who has extensive experience in working with persons with disability in Ghana. The guide was developed in consultation with women with disabilities. All the research team members then reviewed and agreed on the final interview guide.

Majority of the interviews were conducted in the local dialects–*Twi* (in Bosomtwe District), and *Dagbani* and *Gonja* (in Central Gonja District). A few were done in English. Interviews lasted 1 to 1.30 hours. Typically, interviews first captured basic socio-demographic characteristics of participants such as age, level of education, and type of impairment. Interviews then focused on exploring the women’s experiences of pregnancy and childbirth, their desire for children, and their experiences with the health service. Interviews also explored how their disability had affected the maternal healthcare and support they had received in the health facility. Both open and closed questions were asked. For women with speech and hearing impairments, interviews were conducted with the help of the community-based surveillance volunteers, family and friends.

Data were recorded and transcribed, and all non-English transcripts were translated into English. Three independent bilingual specialists each checked the quality of translations from *Twi*, *Dagbani* and *Gonja* to English. All transcripts were exported into Nvivo where coding, categorisation and theme identification were done. Data were analysed using Attride-Stirling’s thematic network analysis framework [24]. The Attride-Stirling thematic network analysis framework provides a technique for breaking up qualitative or textual data, and for performing micro-analysis to show how the structure of talk in field interviews and discussions is connected or disconnected [24]. The framework also allows for open and methodical discovery of emergent concepts and themes and their interconnections [24]. Where appropriate, verbatim quotations from interview transcripts were used to illustrate relevant themes. In reporting the findings, we followed the consolidated criteria for reporting qualitative research (COREQ) [25].

### Ethics

The University of Oxford Social Sciences and Humanities Inter-divisional Research Ethics Committee (Ref No.: SSD/CUREC1/11-051), and the Ghana Health Service Ethical Review Committee (Protocol ID NO: GHS-ERC 18/11/11) gave ethical approval. Informed written and verbal consents were obtained from all participants. Individual participants were requested to sign or thumb print a written informed consent form. Participants (and these were the majority) who could not sign or did not feel comfortable signing the written consent form were permitted to give verbal consent. Each verbal consent process was witnessed by at least one family member or friend. Participation in the study was voluntary, and participants could withdraw any time they wanted to. Confidentiality was maintained throughout the study by using number identifiers on audio recordings, interview notes, and transcripts.

## Results

### Characteristics of participants

In all, 72 women—comprising 47 women with physical impairment, 12 women with hearing impairment, 7 women with visual impairment and 6 women with speech impairment–took part in the study. The youngest participant was 19years and the oldest was 38 years. Majority (54) had no formal education. More than half of the participants (48) had never married, while only four of the participants were either self-employed or worked for some pay.

### Desire for children and experiences with pregnancy and childbirth

Interviews with women with disability suggested that many of them desired to have children. Many reported the joy pregnancy and childbirth brought to them, and noted how pregnancy and childbirth had brought them self-actualisation and empowerment.

I can’t fully describe how happy I was when I became pregnant and finally gave birth safely. I felt fulfilled and accomplished because people now respect me.

Another said:

You don’t know how happy I am today. I really wanted to give birth and now that I’m pregnant I’m very happy.

Others said that they desired to have children because it was only through childbirth they hoped to ensure the perpetuity of their own lineage and secure their future. Indeed, the narratives here suggested that in addition to strong desires to experience parenthood–which is mediated by prevailing social and cultural norms that encourage childbearing especially among married women, fears of future or old age economic insecurity, as well as desire to challenge stigma and negative stereotype about disability, were important drivers of childbirth desire and intention among women with disability. As one participants said:

You see, in our society, people expect women, especially married women to give birth. So if you are a woman and you are properly married and you do not give birth or you are unable to give birth, then it is a problem. That is why I want to give birth.

Another participant also reported:

For me I really want to get pregnant and give birth…I really want a baby boy…somebody who can take care of me when I am old.

One participant also said:

Getting pregnant and giving birth for me is a way of saying that I too can do it. You know in this community, if you have disability, people think that you are not a normal human being…if you are a woman they often think that you cannot or you should not give birth but rather concentrate on your disability. So if I can give birth and raise my child, it will be a way for me to disprove all those who think that I am not a normal person merely because of my disability.

Because most of the women interviewed valued childbirth and desired to have healthy normal children, several of them reported their desire to receive skilled care to ensure safe delivery.

For people like us we need special care during pregnancy to ensure safe delivery. I try to go for antenatal care and I believe every pregnant woman needs it.

### Challenges to maternal healthcare access

Despite the desire for childbirth and skilled care, several of the participants in this study reported how their disability, together with a disability-insensitive organisation and delivery of skilled care services, and other constraints, often make it extremely difficult or impossible for them to access and use such services. Although some participants reported a number of positive experiences, for the purposes of this paper, the challenges they encountered are explored below.

#### Mobility problems

Interviews with the women suggested that mobility from their homes to health facilities to receive care was a major challenge. Most of the women with visual impairment and physical disability as well as those in rural areas particularly reported this challenge, perhaps, because access to maternal healthcare often involves travelling relatively longer distances.

When I was pregnant, I wanted to go to the hospital to see the midwife but because of my condition I couldn’t go.

Mobility problems were often compounded by resource constraints and a public transport system that is not disability-friendly.

I really want to go and check my pregnancy. My problem is how to move from here to the health center. I can’t paddle my wheelchair to the health center because it is far. If I had enough money I will just hire a taxi to take me there…I can’t also use the public bus because I can’t get into the bus and even if I get somebody to help me into the bus where to put my wheelchair is a problem.

Although several women reported that cost of maternal healthcare was not a serious deterrence because of the free maternal healthcare policy, difficulties with mobility and the prohibitive cost involved in arranging appropriate transportation often prevented many women with disability from accessing skilled care.

#### Limited support

The mobility challenge that the women encounter is linked to another problem: limited support from family, community members and the health system. It was widely reported that family and community members tended to be less supportive once a woman with disability got pregnant. According to this account, such women who got pregnant were often chided or reminded of their disability and the need for them to focus on that rather than getting pregnant.

It is not that I don’t want to go for antenatal. My problem is…you know… I can’t move alone without support, and people are very reluctant to help me. They normally say if I knew I couldn’t walk to the clinic then I shouldn’t have gotten pregnant.

Lack of support was particularly pronounced for women with visual disabilities as such people often require the direct assistance of others to access needed healthcare. Similarly, most women with disabilities who were not married reported limited support from family and community members. Educated and married women with disability and those who had children however reported better support systems.

I delivered my first child at home because I didn’t get good support from my family because my husband had travelled and my mother too was ill. But for this second one, I delivered in the hospital because my first child took care of me while I stayed in the hospital.

This notwithstanding, accounts from majority of the women interviewed suggested that negative beliefs and perceptions about disability and reproduction often combine to weaken the support given to women with disability during pregnancy and childbirth.

#### Communication problems

Most women who suffered speech and hearing impairments reported difficulties with communicating with healthcare providers.

Last time I went to the clinic to complain to the midwife about some pains I felt around my waist. But the midwife couldn’t understand what I was talking about…she just gave me paracetamol and asked me to go home.

Another said:

It is frustrating to go to the clinic…nobody seems to understand me. When I go with my husband it is better because he understands me, and he then tells the nurses what I’m saying. But if I go alone, they don’t understand me.

Most of the accounts women gave suggested that many maternal healthcare providers at health facilities could neither understand nor appropriately communicate in sign language. This was a disincentive for women with speech and hearing impairments to seek care at health facilities.

#### Unfriendly healthcare infrastructure

One of the biggest challenges that women with physical disabilities and visual impairment face in accessing skilled care is the unfriendly nature of healthcare infrastructure.

If I go to the hospital now, how to get inside will be a problem. The entrance has a steep staircase that I can’t climb with my wheelchair. Some of the offices too I can’t enter with my wheelchair. It usually takes longer before the nurses will help you enter. The last time I went to check my pregnancy I waited for almost 30 minutes.

One visually impaired woman also reported:

As I can’t see it is always difficult to find my way around, especially if my husband is not around to help me. The health people too, they won’t help you…the last time I went I almost tripped off the stairs. So these are some of the problems I face.

Several women, particularly those suffering physical and visual impairments, reported that most healthcare facilities currently lack ramps, wheelchairs, disability-friendly delivery beds, appropriate separate toilets for disabled persons, and personnel to assist the women climb stairs, examination tables and delivery beds. These problems often combined to discourage some women from seeking skilled care.

#### Healthcare providers’ insensitivity and lack of knowledge

All the women interviewed also reported healthcare providers’ insensitivity to and lack of knowledge of their care needs as a major challenge to their desire to access and use skilled maternal healthcare services.

One time when I was pregnant I went to see the midwife. As soon as I got to the health center one of the nurses shouted to her colleagues ‘come and see a pregnant cripple’. I felt really embarrassed. Since that day I have not been to the health centre.

One woman with physical impairment said:

I think most of the nurses just don’t understand that as a disabled person, we have special needs. The other day I went to see the midwife. She just asked me to lie on the table…but the table was high. When I asked one of the nurses to help me she just started shouting at me…she said why couldn’t I climb and lie down myself. She didn’t seem to understand that I couldn’t stand on my own. She even said if I knew I couldn’t climb the table then I shouldn’t have been pregnant. She also said it wasn’t her job to be helping cripples lie down on tables. I really felt bad.

Some participants also reported that information and advice from healthcare providers were sometimes irrelevant or not applicable to disabled women.

Sometimes the nurses don’t really think about my situation. When I was pregnant and went for checkup, the nurse said I should do exercise by walking every day. But look at me, I can’t stand and walk. I can only move about in this wheelchair. So how do I benefit from this advice?

Several of the accounts here indicated that some healthcare providers were not only rude and insensitive but also they appeared ill-prepared to address the maternity needs of women with disability. Accordingly, most caregivers were not trained to understand disability and to provide care to pregnant women with disability. Indeed, some participants reported that some caregivers were even nervous or uncomfortable examining them.

At times you can tell that the nurses don’t want to help you…sometimes too they are just afraid of you like you’re a lion waiting to eat them up. But it’s not like that. I’m a human being with feelings and so when I go to see the nurse and they treat me like an animal, I won’t go again.

## Discussion

### Summary of findings

The qualitative study reported in this paper is one of the first in Ghana to explore the challenges women with disability encounter in accessing and using institutional maternal healthcare services. Contrary to popular assumptions that these women are either ‘asexual’, ‘passive receivers of help’ or ‘patients’ not capable of marriage or giving birth, our findings suggest that they desire children, are sexually active, and a good number of them have been married. Findings also suggest that although women with disability do want to receive institutional maternal healthcare, they often encounter challenges related to mobility and the use of public transport services as well as access to health facilities. Healthcare providers’ insensitivity and lack of knowledge about the maternity care needs of women with disability, and health information that lacks specificity in terms of addressing the unique maternity care needs of such women, also emerged as a key challenge to these women’s access to maternal healthcare.

### Consistency with previous research

A number of our findings are consistent with previous research. For example, our findings in relation to sexual activity and desire for childbirth among women with disability are consistent with recent evidence that shows that rates of sexual activity and childbirth among women with disability are comparable to those of non-disabled women [26]. Our findings in relation to negative perceptions of women with disability as well as physical inaccessibility of health facilities to women with disability also resonate with one recent study on disability culture in urban Accra [12] and another on the challenges of implementing the World Report on Disability in Ghana [13]. In addition, our findings on healthcare providers’ insensitivity and lack of knowledge about the maternity care needs of women with disability, and health information that lacks specificity in terms of addressing the unique maternity care needs of disabled women, are similar to findings from previous studies on the healthcare access and support for women with disability [7, 9,11, 27]. Indeed, the maternal healthcare needs of women with disability, and the difficulties that these women encounter when accessing institutional maternal healthcare services in Ghana as documented in our qualitative study, are similar to the experiences of women with disability across the world [5, 7–9, 27, 28, 29].

### Explanations for findings and implications

The findings from this study have implications that should not be ignored. Results from the study showed that women with disability are as much sexually active and desire children as their counterparts without disability. In addition to perceptions that pregnancy and childbirth contributed to their self-actualisation, desires to challenge stigma and negative stereotype about disability, and fears of future or old age economic insecurity, were important drivers of childbirth desire and intention among women with disability. The finding that many women with disability desire children, are sexually active, and a good number of them have been married, suggests the need to focus attention on the unique sexual and reproductive healthcare needs and challenges of women with disability in Ghana. In addition to targeting women with disability with modern sexual and reproductive health services such as modern contraception and family planning services, our findings suggest the need to challenge popular but misguided assumptions that such women are either ‘asexual’, ‘passive receivers of help’ or ‘patients’ not capable of marriage or giving birth. This is all the more so because such assumptions have the potential not only to perpetuate stigma against women with disability who are sexually active and desire children, but also to lead to reduced social support and neglect of the maternal and reproductive healthcare needs and challenges of women with disabilities as illustrated in the accounts of a number participants in this study. Rather than implementing community-based rehabilitation programmes, Ghana should start by attempting to adjust these negative perceptions while at the same time creating a physical environment that empowers and enables persons with disabilities to more easily fulfill their social roles [13]. We believe community-based public education on issues of disability as well as the mainstreaming of disability issues into social development and health policies and programmes could help alter some of these misguided assumptions.

The inaccessibility of transport services and healthcare facilities to women with disability is one important challenge participants in this study reported. This inaccessibility is clearly in discord with Ghana’s Disability Act, which calls for access to public places and services for persons with disability as well as integration of their needs into the design, construction and operation of transport network. Such women’s inaccessibility to healthcare facilities constitutes a form of social exclusion and indeed a violation of their right to appropriate and quality healthcare as lucidly illustrated by some previous research in Ghana [6, 30]. This could potentially undermine Ghana’s effort to increase access to healthcare for all through the reduction of physical distance. This suggests the need for existing and future designs of transport facilities, maternity wards and health facilities in general to be made more disability-friendly. For instance, disability-friendly facilities like ramps, wheelchairs, adjustable beds, and specialised sanitary facilities could be provided on public transportation facilities and in health facilities. As mobility is a major access barrier for most of the participants in this study, we also recommend that the Ministry of health and Ghana health service take steps to provide free or subsidised ambulatory or transportation services for women with disability who need or desire to access and use skilled maternal healthcare services at health facilities. This recommendation is indeed similar to that of one recent study, which suggested that the Ghana Health Service should provide a system of mobile health vans to ensure those in rural areas access health care [30]. In addition, incentive schemes could also be instituted for family members or persons who assist women with disability to access skilled care. Such a scheme would be particularly important for women with visual, speech and hearing impairments who often require the direct support of other individuals to effectively access and use skilled maternal and reproductive healthcare services. This could take the form of material incentives such as cash reward or formal national/regional or district level recognition by the state or Ghana health service.

Negative attitudes and practices of healthcare providers toward women with disability is one of the central challenges reported in this paper. Although some of these negative attitudes and practices such as disrespectful care may apply to the general population as shown by some recent studies in Ghana [31, 32], the evidence from this study suggests that they are often conditioned or exacerbated by the disability status of these women, which makes it difficult for them to travel to access care, as well as gain access to rather unfriendly physical healthcare infrastructure. These negative attitudes and practices could be due to lack of health-worker training on issues of disability, erroneous assumptions about the asexuality of women with disability, and misguided assumptions about what such women can or should do. In this regard, there might be the need to fully implement provisions in Ghana’s Disability Act, which enjoins Ghana’s Ministry of Health to include the study of disability and related issues in the curricula of training institutions for health professionals to develop appropriate human resources to provide general and specialised rehabilitation services. In particular, both pre-service and in-service training of health workers must emphasise the principles of patient and family-centred care, as well as customer care communication, especially communication skills in sign language. As some previous studies in Ghana have shown, a focus on training of health personnel on ‘public relations’ could build trust and restore confidence in the healthcare system [31, 32]. Therefore, and as recommended by one recent study, Ghana’s Ministry of Health should ensure that at every district, regional and training hospital there are health professionals that have received detailed training on working with persons with disabilities [30]. Training and education alone might not be enough to address negative attitudes of caregivers. Therefore, effective and supportive leadership, which shows the way forward in terms of decent behaviour towards women with disability, might be required. For this to become a reality, the ‘Code of Patients Rights’ developed by the Ghana Health Service, must be fully and effectively implemented, so that caregivers whose practices promote access to care for women with disability are rewarded, while those who contravene good clinical practice and ethical standard of care, thereby obstructing women’s healthcare seeking, are penalised. This should not be limited to care for women with disability alone; it should be applied both in contexts of care for non-disabled women seeking skilled maternal healthcare services in health facilities and in general healthcare delivery to the entire population as suggested by Ganle and Colleagues [32].

### Strengths and limitations

Together, findings from this study add to a small but growing body of empirical research evidence in low-income settings that highlight the unique maternity care needs of women with disability and the challenges they encounter in accessing and using maternal health services [5, 7, 9, 21, 27–29]. In particular, the qualitative research approach used to document women’s experiences and narrative accounts helped offer pioneering contribution to understanding of the challenges women with disability face in accessing and using maternal healthcare in Ghana, and as well provide opportunity for the healthcare system to take the necessary remedial actions to redress the situation. More importantly, the results provide useful pointers for disability-related organisations and healthcare providers in and outside Ghana to participate in providing technical expertise in the delivery of acceptable healthcare to women with disabilities.

The findings in this study should however be interpreted against a number of limitations. The research was conducted with only 72 women with disability in only two districts. The limitation of applying the findings in other parts of Ghana is therefore acknowledged. Also, the presence of family and friends at interviews with women with speech and hearing impairments could have affected their responses. In addition, our study did not explore the perspective of other stakeholders like healthcare providers. Future research designs could include the perspectives of healthcare providers. Finally, much of the data was self-reported, and collecting data through recall of reproductive history generates information that is liable to recall bias [23]. Moreover, we acknowledge that some meaning may have been lost in the translation of non-English interviews. These limitations notwithstanding, important lessons can be drawn from the findings from this study to inform policies that promote disabled women’s access to and use of skilled maternity care services in other parts of the country and beyond.

## Conclusions

Our study suggests that maternal healthcare services designed to meet the needs of women without disability might lack the flexibility and responsiveness to meet the unique maternity care needs of women with disabilities. If Ghana is to fulfill its international obligations on the right to health for all, as well as attain the maternal health-related Millennium Development Goals, resources must be proactively allocated to support the most vulnerable and underserved segments of the population, including women with disability. Recommendations for change include disability-related cultural competence training for healthcare providers, making healthcare facilities more disability-friendly as well as an emphasis on patient-centred care and behaviour change strategies for healthcare providers and the general public. Interventions related to provision of free or subsidised disability-friendly transport services to address mobility challenges as well as incentive schemes to boost social support for women with disability who need or desire to access and use skilled maternal healthcare services at health facilities, would also be critical. In addition, expedited/fast track/priority service could be provided to women with disability. Also, home visits after first registration or mobile phone follow-ups by healthcare providers could help address challenges related to mobility.

## References

[1] World Health Organization and World Bank. 2011 *World Report on Disability*. Geneva: World Health Organization.

[2] United Nations. 2006 *Convention on the Rights of Persons with Disabilities and Optional Protocol*. Nairobi, Kenya: United Nations.

[3] Hosseinpoor AR, Williams JAS, & Gautam J. 2013 Socioeconomic inequality in disability among adults: a multicountry study using the world health survey. *American Journal of Public Health*, 103: 1273–1286. 10.2105/AJPH.2012.301115 PMC3682610 23678901

[4] Mitra S, Posarac A, & Vick B. 2013 Disability and poverty in developing countries: a multidimensional study. *World Development*, 41: 1–13.

[5] Morrison J, Basnet M, Budhathoki B, Adhikari D, Tumbahangphe K, Manandhar D et al 2014 Disabled women’s maternal and newborn health care in Nepal: A qualitative study. *Midwifery*, 30(2014): 1132–1139. 24768318 10.1016/j.midw.2014.03.012 PMC4217148

[6] Mprah KW, Anafi P, and Sekyere OF. 2014 Does disability matter? Disability in sexual and reproductive health policies and research in Ghana. *International Quarterly of Community Health Education*, 35(1): 21–35. 10.2190/IQ.35.1.c 25416430

[7] Ahumuza SA, Matovu KBJ, Ddamulira JB, & Muhanguzi KF. 2014 Challenges in accessing sexual and reproductive health services by people with physical disabilities in Kampala, Uganda. *Reproductive Health*, 11:59 10.1186/1742-4755-11-59 25086444 PMC4131806

[8] Nosek MA, Young MA, Rintala DH, Howland CA, Foley C & Bennett J. 1995 Barriers to reproductive health maintenance among women with physical disabilities. *Journal of Women’s Health*, 4(5): 505–518.

[9] Trani J, Browne J, Kett M, Bah O, Morlai T, Bailey N et al 2011 Access to health care, reproductive health and disability: a large-scale survey in Sierra Leone. *Social Science & Medicine*. 73(2011): 1477–1489. 22014873 10.1016/j.socscimed.2011.08.040

[10] Mulumba M, Nantaba J, Brolan EC, Ruano LA, Brooker K, & Hammonds R. 2014 Perceptions and experiences of access to public healthcare by people with disabilities and older people in Uganda. *International Journal for Equity in Health*, 13:76 10.1186/s12939-014-0076-4 25928896 PMC4188877

[11] Leigh IW, Poers L, Vash C, & Nettle S. 2004 Survey of psychological services to clients with disabilities: the need for awareness. *Rehabilitation Psychology*, 49:48–54.

[12] Reynolds S. 2010 Disability culture in West Africa: qualitative research indicating barriers and progress in the Greater Accra region of Ghana. *Occupational Therapy International*. 17 (2010) 198–207 10.1002/oti.303 20853313

[13] Tuakli-Wosornu YA, & Haig AJ. 2014 Implementing the World Report on Disability in West Africa: challenges and opportunities for Ghana. *American Journal of Physical Medicine and Rehabilitation*, 93(Suppl): S50–S57. 24356083 10.1097/PHM.0000000000000023

[14] Ghana Statistical Service. 2012 *Ghana Population and Housing Census 2010*. Accra: Ghana Statistical Service.

[15] Persons with Disability Act of Ghana (Act 715), 2006.

[16] World Health Organization. 2014 *Trends in Maternal Mortality*: *1990 to 2013*: *Estimates Developed by WHO*, *UNICEF*, *UNFPA and the World Bank*. Geneva: World Health Organisation.

[17] Ganle JK (2015). Ethnic disparities in utilisation of maternal healthcare services in Ghana: Evidence from the 2007 Ghana maternal health survey. *Ethnicity and Health*, 10.1080/13557858.2015.1015499 25728254

[18] Ghana Statistical Service. 2011 *Ghana Multiple Indicator Cluster Survey with an Enhanced Malaria Module and Biomarker*, *2011*. Accra, Ghana: Ghana Statistical Service.

[19] Witter S, Arhinful KD, Kusi A and Zakariah-Akoto S. 2007 The experiences of Ghana in implementing a user fee exemption policy to provide free delivery care. *Reproductive Health Matters*, 15(30): 61–71. 17938071 10.1016/S0968-8080(07)30325-X

[20] Ganle JK, Parker M, Fitpatrick R, and Otupiri E. 2014 Free maternity care and equity of access to maternal health services in Ghana: a descriptive study. *International Journal for Equity in Health*, 13:89. 25388288 10.1186/s12939-014-0089-z PMC4318433

[21] Aday LA and Anderson R. 1975 *Development of Indices of Access to Medical Care*. Ann Arbour: Health Administration Press.

[22] World Health Organization. 2001 *The International Classification of Functioning*, *Disability and Health*. Geneva: World Health Organization.

[23] Ganle JK, Obeng B, Segbefia YA, Mwinyuri V, Yeboah YJ, & Baatiema L. 2015 How intra-familial decision-making affects women’s access to, and use of maternal healthcare services in Ghana: a qualitative study. *BMC Pregnancy and Childbirth*, 15:173 10.1186/s12884-015-0590-4 26276165 PMC4537557

[24] Attride-Stirling J. 2001 Thematic networks: an analytic tool for qualitative research. *Qualitative Research*, 1(3): 385–405.

[25] Tong A, Sainsbury P, and Craig J. 2007 Consolidated criteria for reporting qualitative research (COREQ): a 32-item checklist for interviews and focus groups. *International Journal of Quality in Health Care*, 19(6): 349–357. 10.1093/intqhc/mzm042 17872937

[26] World Health Organization. 2009 Promoting Sexual and Reproductive Health for Persons with Disabilities *WHO/UNFPA Guidance Notes*. Geneva: World Health Organization.

[27] Becker H, Stuifbergen A, & Tinkle M. 1997 Reproductive health care experiences of women with physical disabilities: a qualitative study. *Archives of medical Physical rehabilitation*, 78:27–33. 10.1016/s0003-9993(97)90218-5 9422004

[28] Bremer K, Cockbum L & Ruth A. 2010 Reproductive health experiences among women with physical disabilities in the Northwest region of Cameroon. *International Journal of Obstetrics and Gynaecology*, 108:211–213. 10.1016/j.ijgo.2009.10.008 19951820

[29] Murthy SVG, John N, Sagar J, & South India Disability Evidence Study group. 2014 Reproductive health of women with and without disabilities in South India, the SIDE study (South India Disability Evidence) study: a case control study. *BMC Women’s Health*, 14:46. 25927587 10.1186/s12905-014-0146-1 PMC4256815

[30] Inclusion Ghana. 2013. Access to health care for persons with intellectual disabilities in Ghana: mapping the issues and reviewing the evidence. Available at: http://inclusionghana.org/resources/reports/Access%20to%20Health%20Care%20for%20Persons%20with%20ID%20in%20Ghana.pdf. Accessed on April 10, 2016.

[31] Ganle JK, Parker M, Fitpatrick R & Otupiri E (2014). A qualitative study of health system barriers to accessibility and utilization of maternal and newborn healthcare services in Ghana after user-fee abolition. *BMC Pregnancy and Childbirth*, 14:425 10.1186/s12884-014-0425-8 25527872 PMC4307897

[32] Ganle JK, Fitzpatrick R, Otupiri E, & Parker M. 2015 Addressing health system barriers to access to and use of skilled delivery services: perspectives from Ghana. *International Journal of Health Planning and Management*, 10.1002/hpm.2291 PMC7614389 25824650

